# New risk score for predicting steroid resistance in patients with focal segmental glomerulosclerosis or minimal change disease

**DOI:** 10.1186/s12014-020-09282-x

**Published:** 2020-05-29

**Authors:** Qinjie Weng, Qiongxiu Zhou, Jun Tong, Yuanmeng Jin, Yunzi Liu, Xialian Yu, Xiaoxia Pan, Hong Ren, Weiming Wang, Jingyuan Xie, Nan Chen

**Affiliations:** 1 Department of Nephrology, Institute of Nephrology, Ruijin Hospital, Shanghai Jiao Tong University, School of Medicine, Shanghai, China; 2 Department of Nephrology, the First Affiliated Hospital of Wenzhou Medical University, Wenzhou, China

**Keywords:** Focal segmental glomerulosclerosis, Minimal change disease, β2-microglobulin, Corticosteroids

## Abstract

**Background:**

Glucocorticosteroid is used for patients with primary nephrotic syndrome. This study aims to identify and validate that biomarkers can be used to predict steroid resistance.

**Methods:**

Our study contained two stages, discovery and validation stage. In discovery stage, we enrolled 51 minimal change disease (MCD) or focal segmental glomerulosclerosis (FSGS) patients treated with full dose steroid. Five urinary biomarkers including β2-microglobulin (β2-MG) and α1-microglobulin (α1-MG) were tested and candidates’ biomarkers were selected based on their associations with steroid response. In validation stage, candidates’ biomarkers were validated in two prospectively enrolled cohorts. Validation cohort A included 157 FSGS/MCD patients. Validation cohort B included 59 membranous nephropathy (MN) patients. Patients were classified into response group (RG) or non-response group (NRG) based on their responses to steroid treatment.

**Results:**

In discovery stage, higher urinary β2-MG was independently associated with response to corticosteroid treatment in MCD/FSGS patients [OR = 1.89, 95% CI 1.02–3.53] after adjusted by age and gender. In validation cohort A, patients in NRG had a significant higher urinary β2-MG [Ln (β2-MG/uCr): 4.6 ± 1.7 vs 3.2 ± 1.5] compared to patients in RG. We then developed a 3-variable risk score in predicting steroid resistance in FSGS/MCD patients based on the best predictive model including Ln(β2-MG/uCr) [OR = 1.76, 95% CI 1.30–2.37], age [OR = 1.005, 95% CI 0.98–1.03] and pathology [MCD vs FSGS, OR = 0.20, 95% CI 0.09–0.46]. The area under the ROC curves of the risk score in predicting steroid response was 0.80 (95% CI 0.65–0.85). However, no such association was found in MN patients.

**Conclusions:**

Our study identified a 3-variable risk score in predicting steroid resistance in patients with FSGS or MCD.

## Background

Primary glomerulonephritis including primary nephrotic syndrome (PNS) is the most common cause of end stage renal disease (PG) in China. Based on pathological changes, common types of PNS include focal segmental glomerulosclerosis (FSGS), minimal change disease (MCD) and membranous nephropathy (MN). The mechanism of PNS is still obscure although some major progresses have been made, such as the findings of PLA2R and THSD7A in MN, Gd-IgA1 in IgAN and podocyte-related genes such as INF2 and APOL1 in FSGS [1–4]. However, no specific agents are available for the treatment of PNS as of today. Therefore, corticosteroids and immunosuppressants are still widely used when massive proteinuria occurs despite the following constraints. First, a significant proportion of these patients show poor responses to the medication. Furthermore, and severe side effects might occur such as infection, metabolic disturbance or osteoporosis [5, 6]. Various risk factors were found to be associated with steroid resistance, including age, abnormal expression of glucocorticoid receptor, mutations of podocyte-related genes, pathological types, abnormal lipid metabolism or immune factors [7]. Given the drawbacks mentioned above, predicting patients’ response before steroid treatment can be very useful. Unfortunately, there is no clinically applicable method to achieve this goal as of now.

Recently, several studies have focused on predictive value of urinary biomarkers to steroid resistance; however, the results of these biomarkers were uncertain and need to be further validated. Five selected biomarkers were illustrated in our study. The first among them were β2-microglobulin (β2-MG), a low-molecular-weight protein (11 kDa) [8, 9] and a single-chain polypeptide consisting of 99 amino acids, a component of human leukocyte antigen (HLA) β chain (light chain) produced by lymphocytes, platelets or polymorphonuclear leukocytes. Hofstra’s study [10] included 57 patients with membranous nephropathy. They found patients with lower urinary β2-MG had a higher remission rate. Therefore, they concluded that urinary β2-MG levels were useful in predicting prognosis. The second biomarker, α1-microglobulin (α1-MG), is another low-molecular-weight protein (26–32 kDa) [11] which is mainly synthesized by liver and lymphocytes. α1-MG is comprised of 167 amino acids and crossreacts with antigen determinants such as HLA. Studies [12] showed that the increase of α1-MG reflected early renal tubulointerstitial injuries. The third urinary biomarker- orosomucoid (ORM), with a molecular weight of approximately 40,000 Da, is mainly produced in the liver in the form of a single-chain polypeptide with five multi-branched N-sugar chains [13]. Previous studies demonstrated that plasma orosomucoid increased in response to inflammation and other stressful stimuli. A few studies [14, 15] showed that urinary excretion of orosomucoid (UOER) was very low in healthy people. Other studies [16, 17] discovered that increased UOER was an independent, powerful predictor of cardiovascular mortality in patients with type 2 diabetes and diabetic nephropathy. The fourth urinary biomarker, yet a frequently detected one is microalbumin (MAU). MAU presenting in patients with type 2 diabetes indicated poorer renal outcomes and increased risk for ESRD [18]. In addition, the urinary microalbumin creatinine ratio is an early and reliable biomarker for renal injury [19, 20]. The last biomarker is retinol binding protein (RBP), a protein with molecular weight of 21,200 Da [21]. RBP consists of a polypeptide chain and a small portion of carbohydrates and it is mainly produced by liver cells and widely distributed in serum, cerebrospinal fluid, urine and other body fluids. RBP was proved to be a biomarker for interstitial fibrosis [22].

This study aims to identify and validate urinary biomarkers that can predict the response to steroid treatment in PNS patients and to establish a risk score to predict steroid response by combine the effect of potential biomarkers and clinical variables.

## Materials and methods

### Study population and design

**Fig. 1:**
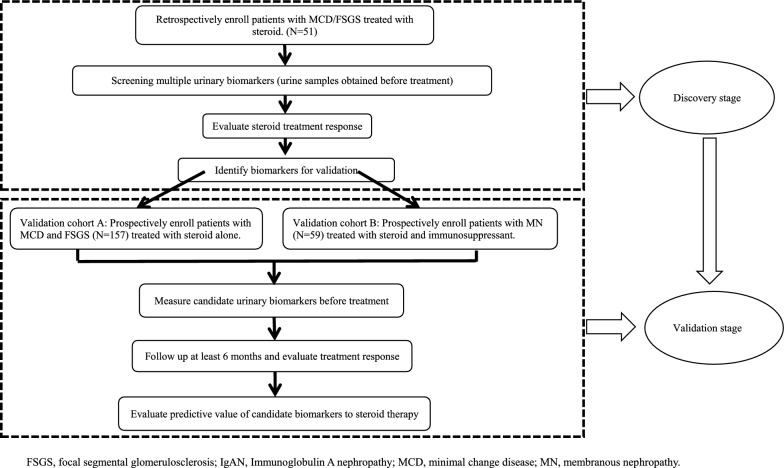
Flow chart of study design

Our study contained two stages (Fig. 1). In the discovery stage, biopsy-proven PNS patients including FSGS or MCD were retrospectively enrolled. The inclusion criteria included PNS patients with biopsy-proven FSGS or MCD; urine protein excretion more than 3.5 g/day; and serum albumin less than 30 g/L. The exclusion criteria were secondary glomerulonephritis such as systemic lupus erythematosus, renal amyloidosis, obesity-related nephropathy, diabetic nephropathy, systemic vasculitis, and human immunodeficiency virus (HIV) related kidney disease; and pregnancy. All patients were given full dosage of prednisone or prednisolone alone [1 mg/(kg/day), maximum to 80 mg/day].

In the validation stage, biopsy-proven PNS patients were enrolled and divided into three cohorts depending on their pathologic diagnosis including FSGS or MCD (validation cohort A) or MN (validation cohort B). The inclusion criteria included patients with biopsy-proven PNS; urine protein excretion more than 3.5 g/day; and serum albumin less than 30 g/L. The exclusion criteria were secondary glomerulonephritis, such as systemic lupus erythematosus, renal amyloidosis, obesity-related nephropathy, diabetic nephropathy, systemic vasculitis, and human immunodeficiency virus (HIV) related kidney disease; and pregnancy. Patients were given full dosage of prednisone/prednisolone alone [1 mg/(kg/day), maximum to 80 mg/day] in validation cohort A or prednisone/prednisolone [0.5–0.8 mg/(kg/day)] combined with immunosuppressants in validation cohort B. Immunosuppressants included cyclophosphamide (0.5 g/m^2^ every month), Cyclosporine (3–4 mg/kg/day), Tacrolimus (0.05 mg/kg/day) or mycophenolate mofetil (1–2 g/day).

The primary outcome of this study was proteinuria remission, which was defined as urine protein decreased by more than 50% compared to baseline and was less than 3.5 g/day after 8 weeks of prednisone/prednisolone treatment. Patients were classified as the response group (RG) or non-response group (NRG) based on whether the primary outcome was achieved.

### Baseline characteristics

Gender, age, body weight and height, systolic blood pressure (SBP), diastolic blood pressure (DBP), creatinine (Scr), urine protein, albumin, body mass index (BMI), uric acid (UA); total cholesterol (TG), and total cholesterol [23] were recorded at the time of renal biopsy. Urine was collected in the morning after at least 9 h’ water prohibition. Urinary α1-MG was measured by rate nephelometry (BNII Protein Analyzer, Siemens, Germany). Urinary β2-MG was measured by chemiluminescent technique (IMMULITE 2000, Siemens, Germany). Urinary ORM and MAU were measured by Immunoturbidimetry (Dako, Denmark). Urinary RBP was measured by Immunoturbidimetry (Byron, Shanghai). Estimated GFR [24] was calculated using the CKD-EPI formula (2009) [25]. Patients were followed up for at least 6 months after recruitment.

### Histological examination

All biopsy specimens were processed by the standard methods for histological examination. Specimens were stained with hematoxylin–eosin, Masson’s trichrome and methenamine silver. Pathologic changes were scored by semiquantitative pathological evaluation with details as below. Global sclerosis was defined as sclerosis involving the entire glomerular tuft. Global sclerosis was scored by the presence of glomeruli with these lesions: 0, absent; 1, present. Segmental sclerosis was defined as tufts involved with sclerosis other than global sclerosis. Segmental sclerosis was scored as followed: 0, absent; 1, present. The severity of interstitial fibrosis, interstitial inflammatory cell infiltration and tubular atrophy were defined as followed: 0, absent; 1, present. The vascular lesion was defined by arterial hyaline change and vascular wall thickening: for either, definitions were 0, absent, or 1, present.

### Statistical analysis

All statistics were performed using SPSS software (version 22). Values are presented as the mean ± standard deviation (SD), unless otherwise stated. As α1-MG and β2-MG levels did not follow a normal distribution, they were normalized by natural logarithm. Independent T test was employed to continuous variables conformed to the normal distribution to evaluate the significance of the difference of mean values between two groups. The variables included age, MAP, BMI, and normal transformed variables including albumin, eGFR, TG, TC, urine protein and β2-MG by natural logarithm. Categorical variables such as pathological evaluation were expressed as frequencies or percentages (%). Pearson Chi square test was applied. We also used logistic regression analysis for evaluating associations between urinary biomarkers and treatment response. In addition, areas under receiver operating characteristic [26] curves were also assessed, and the best diagnostic threshold values were selected. Differences were considered statistically significant with a two-side P value < 0.05.

## Results

### Demographic data and clinical features of patients in the discovery stage

A total of 51 patients were enrolled in the discovery cohort. There were 11 patients with FSGS (21.6%) and 40 with MCD (78.4%). Of all patients, 10 (19.6%) were in the NRG and 41 (81.4%) were in the RG. There were no differences among demographic and clinical variables such as gender, age, urine protein and eGFR between patients from RG and NRG (Table 1).

**Table 1:** Baseline clinical and pathologic characteristics of discovery cohort

Variable	No response	Response	OR (95% CI)^a^
N (%)	10 (19.6)	41 (80.4)	–
Age (year)	38.2 ± 21.9	37.8 ± 19.4	1.001 (0.97–1.04)
Gender (%) male	6 (60.0)	26 (63.4)	1.15 (0.28–4.77)
MAP (mmHg)	99.6 ± 17.6	90.3 ± 18.5	1.05 (0.99–1.12)
BMI (kg/m^2^)	26.2 ± 5.5	24.4 ± 5.1	1.07 (0.93–1.22)
Ln(Pro) (g/24 h)	2.1 ± 0.4	2.0 ± 0.4	1.53 (0.30–7.79)
Albumin (g/L)	17.0 ± 6.7	14.3 ± 5.5	1.10 (0.97–1.25)
eGFR (mL/min/1.73 m^2^)	103.5 ± 20.2	102.3 ± 30.6	1.003 (0.97–1.03)
TG (mmol/L)	3.1 ± 2.2	3.2 ± 1.8	0.98 (0.66–1.47)
TC (mmol/L)	8.5 ± 3.8	10.2 ± 3.1	0.79 (0.60–1.05)
Ln(MAU) (mg/L)	7.9 ± 1.3	8.5 ± 1.2	0.62 (0.34–1.13)
Ln(ORM) (mg/g Cr)	5.0 ± 1.2	5.6 ± 1.0	0.55 (0.27–1.15)
Ln(RBP) (mg/L)	− 1.1 ± 1.7	− 0.7 ± 1.7	0.85 (0.55–1.33)
Ln(β2-MG) (ug/L)	6.6 ± 1.8	5.7 ± 1.4	1.89 (1.02–3.53)^#^
Ln(α1-MG) (mg/dL)	1.6 ± 0.9	1.3 ± 1.0	1.40 (0.63–3.04)
Pathology			
FSGS (%)	5 (45.5)	6 (54.5)	5.83 (1.29–26.46)*
MCD (%)	5 (12.5)	35 (87.5)	0.17 (0.04–0.78)*

MAP: mean arterial pressure; BMI: body mass index; Ln(Pro): natural logarithm of urine protein; eGFR: estimated glomerular filtration rate; TG: triglyceride; TC: total cholesterol; MAU: microalbumin; ORM: orosomucoid; RBP: retinol binding protein; β2-MG: β2-microglobulin; α1-MG: α1-microglobulin; FSGS: focal segmental glomerulosclerosis; IgAN: Immunoglobulin A nephropathy; MCD: minimal change disease

^a^ Clinical characteristics are adjusted by age and gender

*P < 0.05, ^#^P < 0.01

### Screening of urinary biomarkers in the discovery stage

In all patients in the discovery stage, baseline urinary Ln(β2-MG) (6.6 ± 1.8 vs 5.7 ± 1.4) was significantly increased in NRG versus RG (P < 0.01). However, urinary MAU (7.9 ± 1.3 vs 8.5 ± 1.2, P > 0.05), ORM (5.0 ± 1.2 vs 5.6 ± 1.0, P > 0.05), RBP (− 1.1 ± 1.7 vs − 0.7 ± 1.7, P > 0.05) and urinary Ln(α1-MG) (1.6 ± 0.9 vs 1.3 ± 1.0, P > 0.05) showed no difference between the two groups.

### Demographic data and clinico-histological features of patients in the validation stage

In total, 216 biopsy-proven patients were enrolled and divided into two cohorts based on their pathologic diagnosis including 157 FSGS and MCD in validation cohort A and 59 MN in validation cohort B. Patients were given full dosage of prednisone/prednisolone alone [1 mg/(kg/day)] in validation cohort A or prednisone/prednisolone [0.5–0.8 mg/(kg/day)] combined with immunosuppressants in validation cohort B. Among patients from validation cohort B, 24 patients used cyclophosphamide (CTX, 0.5 g/m^2^ every month), 23 patients used Cyclosporine (3–4 mg/kg/day) and eight patients used Tacrolimus (0.05 mg/kg/day). Of all patients in validation cohort A, 54 patients were in NRG (34.4%) and 103 were in RG (65.6%) (Table 2). Baseline eGFR (81.9 ± 37.5 vs 99.1 ± 27.5) (P < 0.01) was significantly decreased in NRG versus RG (OR = 0.98, 95% CI 0.97–0.99). Compared to the RG, patients in the NRG had higher ratios of severe global sclerosis (OR = 2.25, 95% CI 1.11–4.56, P < 0.05), segmental sclerosis (OR = 5.49, 95% CI 2.47–12.17, P < 0.01), interstitial fibrosis (OR = 3.83, 95% CI 1.63–8.97, P < 0.01), inflammatory cell infiltration (OR = 2.20, 95% CI 1.06–4.56, P < 0.05) and tubular atrophy (OR = 2.60, 95% CI 1.22–5.55, P < 0.05) (Table 2).

**Table 2:** Baseline clinical and pathological characteristics of validation cohort A

Clinical variable		No response	Response	OR (95% CI)^a^
N (%)		54 (34.4)	103 (65.6)	–
Age (year)		34.7 ± 14.7	34.3 ± 15.3	1.005 (0.98–1.03)
Gender (male %)		37 (68.5)	60 (58.3)	0.62 (0.31–1.27)
MAP (mmHg)		95.4 ± 16.5	92.7 ± 11.9	1.02 (0.99–1.04)
BMI (kg/m^2^)		24.4 ± 3.7	23.7 ± 3.7	1.04 (0.94–1.14)
Ln(Pro) (g/24 h)		1.8 ± 0.4	1.8 ± 0.4	1.17 (0.52–2.60)
Albumin (g/L)		16.5 ± 6.8	16.1 ± 5.6	1.01 (0.96–1.07)
eGFR (mL/min/1.73 m^2^)		81.9 ± 37.5	99.1 ± 27.5	0.98 (0.97–0.99)^#^
TG (mmol/L)		4.3 ± 3.9	3.6 ± 3.4	1.05 (0.96–1.16)
TC (mmol/L)		10.6 ± 3.6	10.5 ± 4.1	1.001 (0.92–1.09)
Ln(β2-MG/uCr) (ug/mmoL)		4.6 ± 1.7	3.2 ± 1.5	1.76 (1.30–2.37)^#^

Pathological evaluation	Score	No response	Response	OR (95% CI)
FSGS (%)		46 (45.5)	55 (54.5)	5.02(2.16–11.7)^#^
MCD (%)		8 (14.3)	48 (85.7)	0.20(0.09–0.46)^#^
Global sclerosis (%)	0	27 (50.0)	74 (71.8)	-ref-
	1	23 (42.6)	28 (27.2)	2.25(1.11–4.56)*
Segmental sclerosis (%)	0	10 (18.5)	59 (57.3)	-ref-
	1	40 (74.1)	43 (41.7)	5.49(2.47–12.17)^#^
Interstitial fibrosis (%)	0	8 (14.8)	43 (35.0)	-ref-
	1	42 (77.8)	59 (57.3)	3.83(1.63–8.97)^#^
Inflammatory cell infiltration (%)	0	14 (25.9)	47 (45.6)	-ref-
	1	36 (66.7)	55 (53.4)	2.20(1.06-–4.56)*
Tubular atrophy (%)	0	12 (22.2)	46 (44.7)	-ref-
	1	38 (70.4)	56 (54.4)	2.60(1.22–5.55)*
Vascular lesion (%)	0	29 (53.7)	62 (60.2)	-ref-
	1	20 (37.0)	40 (38.8)	1.07 (0.53–2.14)

MAP: mean arterial pressure; BMI: body mass index; Ln(Pro): natural logarithm of urine protein; eGFR: estimated glomerular filtration rate; TG: triglyceride; TC: total cholesterol; β2-MG: β2-microglobulin; α1-MG: α1-microglobulin; uCr: urinary creatinine; FSGS: focal segmental glomerulosclerosis; MCD: minimal change disease; IgAN: Immunoglobulin A nephropathy; MN: membranous nephropathy

^a^ Adjusted by age and gender

*P < 0.05, ^#^P < 0.01

### Validating of urinary β2-MG with response to steroid treatment in the validation stage

Then we validate the urinary β2-MG in the validation cohort. We confirmed baseline urinary Ln(β2-MG/uCr) (4.6 ± 1.7 vs 3.2 ± 1.5, P < 0.01) was significantly increased in NRG versus RG in validation cohort A. Univariate logistic regression analysis showed higher urinary Ln(β2-MG/uCr) (OR = 1.76, 95% CI 1.30–2.37, P < 0.01) was associated with higher risk for steroid resistance in FSGS and MCD patients (Table 2). Even after fully adjusting for age, gender, clinical parameters and pathological parameters (segmental sclerosis, interstitial fibrosis, inflammatory cell infiltration and tubular atrophy), higher urinary Ln(β2-MG/uCr) (OR = 1.51, 95% CI 1.09–2.08, P < 0.01) was found to be independently associated with steroid resistance in FSGS and MCD patients. However, the associations between Ln(β2-MG/uCr) and steroid resistance were not validated in patients with MN (validation cohort B) (OR = 1.51, 95% CI 0.36–6.38) (Tables 3 and 4).

**Table 3:** Baseline clinical and pathological characteristics of validation cohort B

Clinical variable		No response	Response	OR (95% CI)^a^
N (%)		25 (42.4)	34 (57.6)	–
Age (year)		49.7 ± 17.4	59.9 ± 14.7	0.96 (0.93–0.995)*
Gender (male %)		19 (76.0)	20 (58.8)	0.45 (0.14–1.42)
MAP (mmHg)		103.8 ± 14.0	101.8 ± 12.4	1.01 (0.97–1.05)
BMI (kg/m^2^)		25.6 ± 3.0	24.7 ± 3.2	1.11 (0.93–1.32)
Ln(Pro) (g/24 h)		2.0 ± 0.4	1.8 ± 0.4	2.76 (0.75–10.16)
Albumin (g/L)		19.0 ± 4.7	18.3 ± 4.1	1.04 (0.92–1.17)
eGFR (mL/min/1.73 m^2^)		91.1 ± 24.6	87.3 ± 28.1	1.01 (0.99–1.03)
TG (mmol/L)		2.8 ± 1.3	3.5 ± 3.6	0.91 (0.72–1.14)
TC (mmol/L)		7.9 ± 2.9	8.0 ± 1.4	0.96 (0.75–1.23)
Ln(β2-MG/uCr) (ug/mmol)		3.8 ± 2.2	3.4 ± 1.5	1.15 (0.84–1.57)

Pathological evaluation	Score	No response	Response	OR (95% CI)
MN (%)		25	34	
Global sclerosis (%)	0	13 (52.0)	9 (26.5)	-ref-
	1	12 (48.0)	25 (73.5)	0.33 (0.11–0.99)
Segmental sclerosis (%)	0	23 (92.0)	31 (91.2)	-ref-
	1	2 (8.0)	3 (8.8)	0.90 (0.14–5.82)
Interstitial fibrosis (%)	0	3 (12.0)	3 (8.8)	-ref-
	1	22 (88.0)	31 (91.2)	0.71 (0.13–3.85)
Inflammatory cell infiltration (%)	0	1 (4.0)	4 (11.8)	-ref-
	1	24 (96.0)	30 (88.2)	3.20 (0.33–30.55)
Tubular atrophy (%)	0	3 (12.0)	4 (11.8)	-ref-
	1	22 (88.0)	30 (88.2)	0.98(0.20–4.82)
Vascular lesion (%)	0	10 (40.0)	7 (20.6)	-ref-
	1	15 (60.0)	27 (79.4)	0.39 (0.12–1.23)

MAP: mean arterial pressure; BMI: body mass index; Ln(Pro): natural logarithm of urine protein; eGFR: estimated glomerular filtration rate; TG: triglyceride; TC: total cholesterol; β2-MG: β2-microglobulin; α1-MG: α1-microglobulin; uCr: urinary creatinine; FSGS: focal segmental glomerulosclerosis; MCD: minimal change disease; IgAN: Immunoglobulin A nephropathy; MN: membranous nephropathy

^a^ Adjusted by age and gender

*P < 0.05, ^#^P < 0.01

**Table 4:** Logistic regression of urinary Ln(β2-MG/uCr) with response to steroid treatment in validation cohort A and B

	OR (95% CI)^a^	OR (95% CI)^b^	OR (95% CI)^c^
Validation cohort A	1.62 (1.18–2.21)^#^	1.58 (1.15–2.15)^#^	1.51 (1.09–2.08)*
Validation cohort B	1.96 (0.54–7.25)	2.32 (0.63–8.52)	1.51 (0.36–6.38)

Validation cohort A: FSGS + MCD (N = 157)

Validation cohort B: MN (N = 59)

Renal outcome defined as no response to therapy

β2-microglobulin; uCr: urinary creatinine

^a^ Adjusted by age, gender and clinical parameters (eGFR)

^b^ Adjusted by age, gender and pathological parameters (global sclerosis, segmental sclerosis, interstitial fibrosis, inflammatory cell infiltration and tubular atrophy)

^c^ Adjusted by age, gender, clinical parameters (eGFR) and pathological parameters (global sclerosis, segmental sclerosis, interstitial fibrosis, inflammatory cell infiltration and tubular atrophy)

*P < 0.05, ^#^P < 0.01

**Fig. 2:**
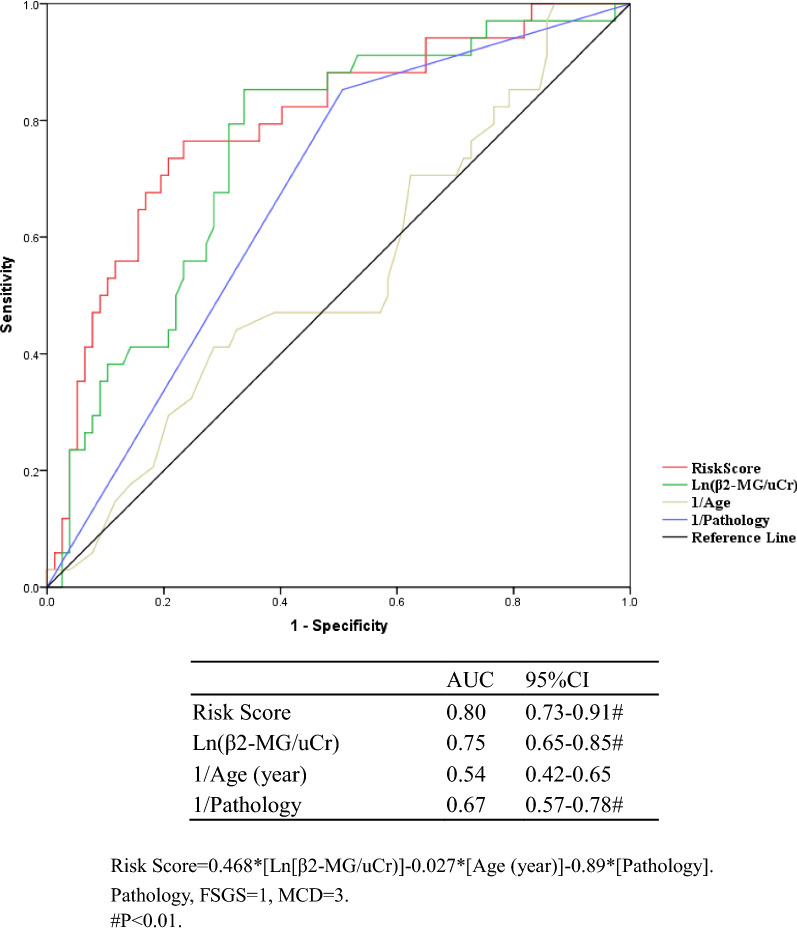
ROC curves of predicting steroid resistance in validation cohort A (N = 157)

In the multivariate stepwise logistic regression model, the best model in predicting steroid resistance included three baseline variables (Fig. 2). These included: Ln(β2-MG/uCr) [OR = 1.76, 95% CI 1.30–2.37], age [OR = 1.005, 95% CI 0.98–1.03], pathology (defined as FSGS = 1, MCD = 3) [OR = 0.20, 95% CI 0.09–0.46]. Based on this model, we developed a 3-variable equation to calculate risk score for predicting steroid resistance in FSGS/MCD patients. The discrimination of the risk score was estimated by AUC (the area under the ROC curves) in the validation cohort A [0.80 (0.65–0.85)], indicating good discrimination (Fig. 2).

**Fig. 3:**
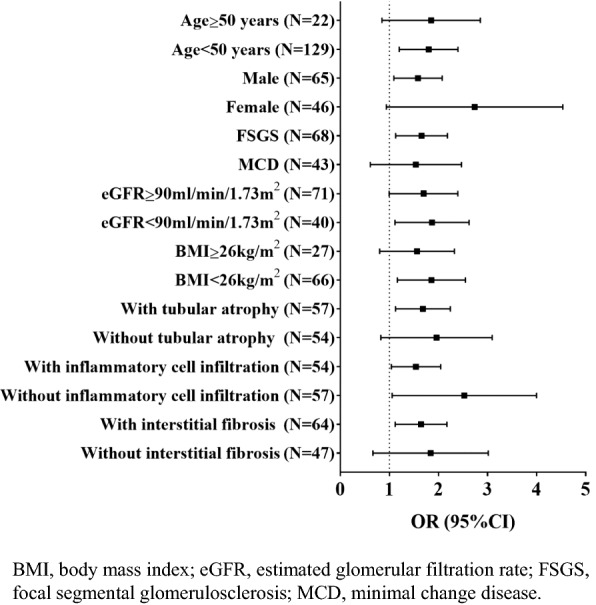
Effect of urinary β2-MG in subgroups in validation cohort A

Furthermore, the associations between urinary Ln(β2-MG/uCr) and steroid resistance were robust in subgroups analysis, including eGFR and some pathological parameters subgroups, except in elderly and overweight patients (Fig. 3). We divided patients into two tertile groups according to their urinary β2-MG value. Clinico-histological features and response to steroid treatment were compared among two groups. Compared to patients in the low tertile group, patients in the high tertile group had lower eGFR and higher levels of TG and tubular-interstitial lesion. No response rate was significantly higher in patients from the high tertile group (OR = 11.38, 95% CI 3.94–32.84, P < 0.01) compared with patients from the low tertile group (Table 5).

**Table 5:** Baseline clinical characteristics of patients in different β2-MG groups in validation cohort A

Ln(β2-MG/uCr)	Low tertile (≤ 2.45)	High tertile (> 2.45)	
Variable	N = 56	N = 55	OR (95% CI)
No response (%)	5 (8.9)	29 (52.7)	11.38 (3.94–32.84)^#^
Age (year)	33.0 ± 14.2	38.0 ± 15.9	1.02 (0.997–1.05)
Gender (%) male	33 (58.9)	32 (58.2)	1.03 (0.49–2.20)
MAP (mmHg)	92.1 ± 10.8	94.3 ± 18.9	1.01 (0.99–1.04)
BMI (kg/m^2^)	23.7 ± 3.8	24.9 ± 3.9	1.08 (0.97–1.21)
Ln(Pro) (g/24 h)	1.8 ± 0.4	1.8 ± 0.4	1.06 (0.45–2.46)
Albumin (g/L)	16.5 ± 5.2	16.4 ± 7.0	0.996 (0.94–1.06)
eGFR (mL/min/1.73 m^2^)	100.9 ± 23.5	86.0 ± 39.0	0.99 (0.97–0.998)*
TG (mmol/L)	2.9 ± 2.4	4.4 ± 4.2	1.20 (1.002–1.44)*
TC (mmol/L)	10.0 ± 3.7	11.1 ± 4.9	1.06 (0.97–1.17)
Interstitial fibrosis (%)	25 (44.6)	39 (70.9)	3.02 (1.387–6.63)^#^
Inflammatory cell infiltration (%)	21 (37.5)	33 (60.0)	2.50 (1.16–5.37)*
Tubular atrophy (%)	24 (42.9)	33 (60.0)	2.00 (0.94–4.26)

MAP: mean arterial pressure; BMI: body mass index; Ln(Pro): natural logarithm of urine protein; eGFR: estimated glomerular filtration rate; TG: triglyceride; TC: total cholesterol; MAU: microalbumin; ORM: orosomucoid; RBP: retinol binding protein; β2-MG: β2-microglobulin; α1-MG: α1-microglobulin

*P < 0.05, ^#^P < 0.01

## Discussion

Corticosteroids remain to be used as the basic treatment of PNS patients in spite of the emerging treatments and drugs in recent years. Past clinical practice of steroid application has indicated that steroid treatment has been proved to be effective for some PNS patients especially those with MCD/FSGS, whereas other patients are steroid resistance. Of those patients who respond to steroid at the initial stage of treatment, some cease to respond effectively as the disease progresses. The fact that PNS patients respond differently to steroid therapy has raised the concern that serious complications resulted from large doses of steroid usage could have happened if patients were to show steroid resistance during treatment. Unfortunately, clinicians are still unable to predict steroid resistance before treatment. Therefore, identification of risk factors for steroid resistance will be essential to clinical practice to reduce the side effects of unnecessary corticosteroid therapy. In this study, we first tested five urinary biomarkers from both FSGS and MCD patients in the discovery cohort and then recorded and identified their associations with steroid resistance. One candidate urine biomarker, urinary β2-MG, was selected and validated in the three validation cohorts. In summary, we identified and validated that urinary β2-MG could be used as an early biomarker for steroid resistance in patients with FSGS or MCD.

A previous study [5] showed that steroid resistance was related to the renal pathological types of patients. For instance, most MCD patients are steroid sensitive, while approximately 30–50% of FSGS patients are steroid resistant [6]. In our study, we found that 42.3% FSGS patients presented with steroid resistance, higher than MCD patients (21.4%). We therefore concluded that the data was aligned with that of the previous study. Increasing number of studies have shown that certain biomarkers could be utilized as important reference for clinical work because of their potential values in predicting the effect of steroid therapy and prognosis of PNS. However, the conclusions of these studies still raise concerns given the constraints that no systematic investigation of different urinary biomarkers was conducted and most of the study results lacked external validation. Our study investigated and validated the predictive value of a series of urinary biomarkers in PNS patients. In the discovery stage, we found that baseline urinary β2-MG significantly increased in FSGS and MCD patients in the steroid resistant group versus those in the steroid sensitive group. Then, the finding was validated in a prospectively collected FSGS and MCD cohort, and an independent association between higher urinary β2-MG and steroid resistance was successfully validated. No such relationship was found in MN patients.

β2-microglobulin, a light-chain molecule of the major histocompatibility complex (MHC) class I antigens, is one of these proteins we screened. β2-microglobulin is a low-molecular-weight protein that is found on the surface membrane of almost all nucleated cells. β2-microglobulin is freely filtered by the glomerular filtration barrier and reabsorbed by the renal tubular cells. β2-MG levels increase in patients with diseases such as renal tubulointerstitial injuries, autoimmune disease and multiple myeloma [27]. Previous studies showed that the level of urinary β2-MG and *N*-acetyl-beta-d-glucosaminidase (NAG) associated with interstitial-tubular lesions were elevated in steroid resistant patients. In the study of Fede et al. [28], urinary NAG and β2-MG levels were assessed in 19 patients with nephrotic syndrome (NS) and in 30 healthy controls. Results showed that steroid resistant patients had significant higher levels of baseline urinary NAG and β2-MG levels than steroid sensitive patients and healthy controls. In the study of Vallés et al. [29], 34 patients with NS were enrolled. Urinary β2-MG level was significantly increased in patients with steroid resistant NS (SRNS) compared to patients with steroid dependent NS (SDNS) or steroid sensitive NS (SSNS). There were no differences between the SRNS group and SSNS in relapse. Urinary NAG level was significantly higher in patients in the SRNS group than those in the SDNS, SSNS, and control groups. However, it was still unclear whether the association with β2-MG, NAG and steroid resistance was independent of interstitial-tubular lesions. Sesso et al. [30] enrolled 37 patients with the nephrotic syndrome. They suggested β2-MG levels could help identify patients more sensitive to steroids. Although several studies can be found about urinary β2-MG and steroid resistance in renal diseases, none of these studies focused on FSGS and MCD which had unique pathogenesis compared to other kidney diseases. In addition, limited sample size restrained these studies from excluding the effects of other confounding factors such as tubulointerstitial lesions. In the present study, we found urinary β2-MG level of FSGS and MCD patients was associated with interstitial fibrosis, inflammatory cell infiltration and tubular atrophy, and increased with the severity of renal tubular injury. Nonetheless, the association between urinary β2-MG and steroid response was robust after adjusting for demographic, clinical and histological variables including segmental sclerosis, interstitial fibrosis, inflammatory cell infiltration and tubular atrophy. Furthermore, subgroup analysis showed urinary β2-MG level was significantly associated with steroid resistance. The conclusion still held true, even for patients without inflammatory cell infiltration. Therefore, our data indicates that the association between urinary β2-MG and corticosteroids response not only reflects tubulointerstitial injury but also suggests an additional mechanism linked to steroid resistance.

β2-MG levels are mainly determined by turnover and activity of lymphocytes in lymphoproliferative and autoimmune diseases [26, 31, 32] such as in Sjögren’s syndrome and SLE. Prior to recognizing the important role that antiphospholipase A2 receptor (anti-PLA2R) autoantibodies played in primary MN, consistent data from studies in animals had already shown that antibody produced by autoreactive B cell clones initiated the events that eventually led to injury to the glomerular filtration barrier and consequent proteinuria [2, 33–35]. The data above suggested that activation of B cells might result in the increase of serum β2-MG level. Other studies had shown that increase of serum β2-MG level would be the consequence of an enhanced surface expression and shedding of MHC class I molecules, mainly on activated macrophages induced by various cytokines (IFN-γ, TNF-α, etc.) [31]. These studies implied that activation of B cells and macrophages might contribute to the increase of serum β2-MG level. Further research is needed to clarify the mechanism.

We acknowledge that this study has limitations. First, in the discovery stage, although urine was collected in the morning after water prohibition, urine proteins were not adjusted by urinary creatinine. Second, multicenter clinical trials with large sample sizes are necessary to validate our findings given the limitation of our single center study. Lastly, combined effects of multiple biomarkers may better enhance the creditability and accuracy in predicting corticosteroid response than the effect of one single biomarker in our study.

## Conclusions

In short, our study screened five urinary biomarkers PNS patients. We identified and validated that urinary β2-MG could independently predict the response to corticosteroid treatment. We developed a 3-variable risk score in predicting steroid resistance in patients with FSGS or MCD. Our results are helpful for clinicians to avoid the abuse of steroid usage in these patients to decrease unnecessary side effects.

## Data Availability

The datasets used and analyzed during the current study are available from the corresponding author on reasonable request.

## Abbreviations

FSGS: Focal segmental glomerulosclerosis
IgAN: Immunoglobulin A nephropathy
MCD: Minimal change disease
RG: Response group
NRG: Non-response group
MAP: Mean arterial pressure
BMI: Body mass index
Ln(Pro): Natural logarithm of urine protein
eGFR: Estimated glomerular filtration rate
TG: Triglyceride
TC: Total cholesterol
MAU: Microalbumin
ORM: Orosomucoid
RBP: Retinol binding protein
β2-MG: β2-Microglobulin
α1-MG: α1-Microglobulin
